# Quantification of global mitochondrial DNA methylation levels and inverse correlation with age at two CpG sites

**DOI:** 10.18632/aging.100892

**Published:** 2016-02-17

**Authors:** Shakhawan K. Mawlood, Lynn Dennany, Nigel Watson, John Dempster, Benjamin S. Pickard

**Affiliations:** ^1^ Strathclyde Institute of Pharmacy and Biomedical Sciences, University of Strathclyde, Glasgow, Scotland, UK; ^2^ Centre for Forensic Science, Department of Pure and Applied Chemistry, University of Strathclyde, Glasgow, Scotland, UK

**Keywords:** mitochondrial DNA, epigenetics, ageing, age prediction, Illumina NGS

## Abstract

Mammalian ageing features biological attrition evident at cellular, genetic and epigenetic levels. Mutation of mitochondrial DNA, and nuclear DNA methylation changes are well established correlates of ageing. The methylation of mitochondrial DNA (mtDNA) is a new and incompletely described phenomenon with unknown biological control and significance. Here we describe the bisulphite sequencing of mtDNA from 82 individuals aged 18‐91 years. We detected low and variable levels of mtDNA methylation at 54 of 133 CpG sites interrogated. Regression analysis of methylation levels at two CpG sites (M1215 and M1313) located within the 12S ribosomal RNA gene showed an inverse correlation with subject age suggesting their utility as epigenetic markers of ageing.

## INTRODUCTION

Quantifying the nuclear DNA (nDNA) methylation of cytosine bases at specific autosomal genetic loci has been widely used to estimate an individual's age for forensic and medical purposes [1-5]. The existence of methylated cytosines within mtDNA has been controversial. Originally proposed four decades ago, the presence of mitochondrial methylation has been difficult to confirm [6, 7]. Maekawa *et al.* showed signals for mitochondrial methylation in 2004, [8] and Infantino and co-workers provided the first evidence of methylated bases (5-methyl-2′-deoxycytidine) present in human mtDNA using mass spectrometry[9]. MtDNA methylation has now been reported in the form of methylcytosine (mC) and hydroxymethylcytosine (hmC) modification[10], and a mitochondrially targeted DNA methyltransferase 1 enzyme (mtDNMT1) is suggested to be responsible[11, 12, 13, 14-18].

MtDNA methylation has been proposed as a cause of ageing and disease [13, 19, 20]. A comprehensive profile of methylation levels across the mitochondrial genome and across ages would be an invaluable starting-point to address the role of epigenetics in these biological phenomena. Here, we describe the next generation sequencing analysis of blood-derived, bisulphite-treated mtDNA taken from a cohort of subjects with ages ranging from 18 to 91, using the Illumina MiSeq sequencing platform.

We selected functional regions of interest (ROI) within the mitochondrial genome and designed primers for methylation analysis – for example, the displacement loop (D-loop)/hypervariable regions that contain the promoters for the ‘heavy’ and ‘light’ strand polycistronic mRNAs, specific mitochondrial genes, and regions where published evidence already exists for methylation. [12] In addition, two replicated sites of mtDNA mutation were chosen because of their potential to create or destroy CpG dinucleotides: an A11778G associated with Leber`s Heriditary Optic Neuropathy, [21] and a T8993G mutation, which is associated with a neurological phenotype [22, 23].

## RESULTS

The mitochondrial genome sequence (GRCH38 or Anderson/Cambridge reference sequence) [24] consists of 16,565 bp in which 435 predicted CpG sites are present (Supplementary 1). We used bisulphite treatment and high-throughput sequencing (Illumina) to assess methylation levels within regions of interest chosen because of their functional importance within the mitochondrial genome. In total the study queried methylation levels at 133/435 CpG sites in the first sample set of DNA from 41 individuals.

**Figure 1:**
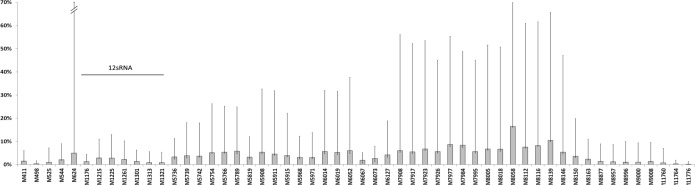
Mean methylation levels at 54 CpG sites across the mitochondrial genome (category labels denote reference base position). While methylation levels are typically 2‐6%, considerable inter‐individual variation was observed, indicated by minimum and maximum range bars. CpG sites within the 12S RNA gene are highlighted.

We identified unambiguous evidence for non-zero mtDNA methylation at 54 of the 133 CpG sites sequenced with average read depth of 2000 (Fig. 1). Methylation levels were, on average, very low (often between 2-6%) but showed regional differences across the mitochondrial genome and, more importantly, a great variance between individuals, as the methylation maxima and averages indicate (Fig. 1).

The correlation between methylation and age was determined in the data set sample using Pearson's correlation coefficient for the 54 sites. Sites MT1215 and MT1313, both in the 12S RNA gene (*MT-RNR1*) showed significant methylation changes (hypomethylation) with increasing age (MT1215, R=-0.322 p=0.043. mt1313 R=- 0.383, p=0.015). These findings were consistent with age-related changes reported in the general 12S RNA gene region by others [13, 26].

**Figure 2:**
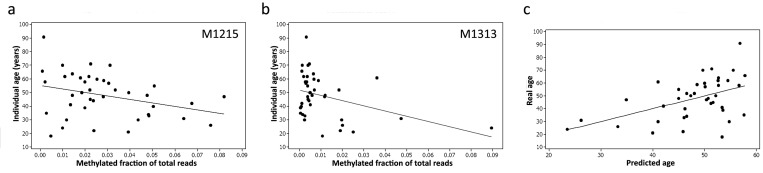
Methylation levels (expressed as a fraction) at two CpG sites within the 12S RNA gene, M1215 (**a**) and M1313 (**b**), correlate with age and can be used to construct an accurate predictive model (**c**) using Pearson's correlation.

In the next step, multivariate linear regression was applied to the methylation data from both M1215 and M1313 (Fig. 2a, b) and a linear regression prediction model was developed relating age to methylation.

The prediction accuracy of the model and contribution of particular predictors were assessed using the adjusted R^2^ statistic, which measures the proportion of age variation explained by the developed model. The model yielded an R^2^=0.509 indicating that 50% of the variation in methylation could be explained by age. The mean absolute deviation (MAD) between predicted and chronological age was 9.3 years (Fig. 2c).

The second sequenced library dataset (n=41 individuals) was used to evaluate the model. From this set, 31 samples (64.5%) had ages correctly predicted to within a ± MAD. The analysed samples were also divided into three age categories: I: 18–39 years, II: 40–59 years, III: 60 years and older. Prediction results were significantly better for samples from category II (83.3% correct predictions) than for categories I (53.8%) and III (20% correct predictions), suggesting that innate or environmental methylation-determining factors, in addition to age, are at play in early and late life.

## DISCUSSION

Although previous reports have shown correlation of mtDNA damage and mutation with aging, [27] it was only very recently that studies suggested that mitochondrial methylation could be used as an ageing biomarker [13, 15, 28]. Dzitoyeva and co-workers have reported the existence of mitochondrial DNA (hydroxy)methylation and its increase with ageing in subregions the mouse brain.[10] Our study provides the most comprehensive map of methylation levels at 133 of the available 435 CpG sites in the mitochondrial genome from 82 individuals. Quantified changes in the methylation state (hypomethylation) of two 12S RNA gene CpG sites (M1215 and M1313) correlate with age. This novel discovery opens new avenues to investigate the mtDNA changes associated with health status and in the processes of criminal investigation. The CpG pair at M1215 and M1313 could only have been detected using high throughput sequencing, such as by Illumina MiSeq300 v2. Therefore, such sensitive and advanced methodologies can solve the issue of failure to detect low-level methylation in the mtDNA genome that may explain reported absence of mitoepigenetics [29, 30], its failure as a health tool marker [8] or its description as having an unusual CpG pattern.[14] Also contributing to this confusion might have been the use of quantification techniques such as ELISA and MeDIP that may struggle with low-level methylation.

The correlation between M1215/M1313 methylation and age was especially accurate for younger and middle-aged individuals in contrast to the poorer predictive power after the age of 60. This might reflect differences in individual lifetime metabolic health or exposure to environmental influences, thus giving rise to discrepancies between biological and chronological age. Furthermore, we speculate that these lifeaccumulated environmental influences cause the substantial general mtDNA methylation variance observed between individuals. Alternatively, methylation differences might also reflect varying representation of cell-types in blood samples – again, a potential surrogate for cytological investigation that might provide useful insights into immunological status. Our study did not differentiate between 5mC and 5hmC as the chemical conversion by sodium bisulphite (the gold standard detection technique for methylation studies) distinguishes only converted cytosines from non-methylated cytosines. The study has some limitations that have been taken into account in data interpretation. Further studies are needed to isolate mtDNA and analyse its methylation in specific subtypes of blood cells, such as platelet progenitors and lymphocytes. Further, mitochondrial DNA analysis from other populations and from male samples needs to be carried out to assess the generality of our findings. Lastly, the impact on mtDNA methylation of determinants of ill health, including smoking, pollution, obesity and chronic life stress should be studied to quantify external influences.

To conclude, Illumina sequencing of 82 human blood samples indicated clear methylation patterns in mtDNA at 54 CpG sites in mitochondrial genome and that the level of the methylation was variable among different ages (18-91 years). Two of these CpG sites (M1215 and M1313) showed stronger correlation between predicted and chronological age with only about 9 years mean absolute difference (MAD). This finding implies that mtDNA methylation will be an available biological marker for forensic age-prediction and health status measurement.

## PATIENTS, MATERIALS AND METHODS

### Samples

The project was approved by the University of Strathclyde Ethics Committee and, prior to sample collection, participants signed an informed consent statement. A qualified phlebotomist took 5ml blood samples from 82 female volunteers (age 18-91 yrs) at Erbil Hospital, Iraq. All the volunteers were from the Kurdish ethnic group but were recruited randomly without any health history or background information on education, marriage status, BMI, smoking, parity or http://www.impactaging.com 638 AGING, April 2016, Vol. 8 No.4 lifestyle. 100 μL aliquots were immediately placed in Eppendorf tubes and DNA extracted. The extracted DNA was quantified by Stratagene 3005X qPCR instrument (Agilent Technologies, CA, USA), using the Quantifiler® Human DNA Quantification Kit cat. # 4343895 (Life Technologies, CA, USA).

### Region of interest selection

The mitochondrial sequence (GRCH38 or Anderson/Cambridge reference sequence) [24] consists of 16,565 bp in which 435 predicted CpG sites are present (Supplementary 1). Regions of interest (ROI) were chosen on the basis of biological function: the promoters for both ‘heavy’ and ‘light’ strand of the D-loop regions, polycistronic mRNAs and most of the functional genes of mitochondria. In addition, two replicated sites site of mtDNA mutation were chosen because of their potential to create or destroy CpG dinucleotide: A11778G associated with Leber`s Heriditary Optic Neuropathy, [21] and a T8993G mutation, which is associated with a neurological phenotypes [22, 23]. Primer sequences used to amplify the RsOI (each amplicon 100-300bp in length) and the amplicon sequence (before and after bisulphite conversion) are in Supplementary 2. Primers targeting 10 ROI amplicons (Table 1) were designed using Methyl Primer Express Software v1.0 (Applied Biosystem, Foster City, California), synthesised (Applied Biosystems, Foster City, California, and Life Technologies, CA, USA), and successfully passed amplification quality control criteria. Some locations within each ROI were impossible to amplify due to repeat sequences or high CpG content.

**Table 1:** 10 Amplicons which passed Illumina quality control criteria. The location of each ROI in mitochondria genome (ChrM) and estimated amplicon number in the mDNA are shown as well

ROI	Location	Estimated number of amplicons
Promoter (+)	ChrM:141-700	1
Promoter (−)	ChrM:544,525-624	1
12s Ribosomal RNA	ChrM:501-1700	1
Origin of light strand	ChrM:5541-6040	1
Cytochrome C oxidative subunit1	ChrM:5541-6040	1
Cytochrome C oxidative subunit2	ChrM:5781-6340	2
Cytochrome C oxidative subunit3	ChrM:=9141-9940	1
MT-ND6	ChrM:=14001-14740	1
T8993G	ChrM:=8781-9160	1
G11778A	ChrM:11541-12040	1
Total		10

### Bisulphite analysis and library construction

The mtDNA was modified (non-methylated C residues converted to U) via a bisulphite conversion step, EZ DNA Methylation-DirectTM Kit (Cat. 5020 and 5021, Zymo Research).[25] Multiplex PCR amplification of the RsOI (Fluidigm Access ArrayTM System, BioMark, USA) generated pools of amplicons that also employed a universal forward tag common sequence 1 (CS1), and universal reverse tag common sequence 2 (CS2) (CS1= 5′-ACACTGACGACATGGTTCTACA-3′, CS2 = 5′-TACGGTAGCAGAGACTTGGTCT-3′). Each individual amplicon pool was subsequently barcoded. Two age-matched libraries, each consisting of 41 individuals, were created and purified (ZR-96 DNA Clean & Concentrator™ - ZR, Cat.# D4023) and then prepared for massively parallel sequence by Illumina next generation sequencing (NGS) using a MiSeq V2 300bp Reagent Kit (cat. # MS-102-2001), (paired-end sequencing protocol) according to the manufacturer's guidelines.

## SUPPLEMENTARY DATA







## References

[1] Yi SH, Xu LC, Mei K, Yang RZ, Huang DX (2014). Isolation and identification of age-related DNA methylation markers for forensic age-prediction. Forensic Science International: Genetics.

[2] Burgess DJ (2012). Human epigenetics: Showing your age. Nature Reviews Genetics.

[3] Bocklandt S, Lin W, Sehl M, Sánchez F, Sinsheimer J (2011). Epigenetic Predictor of Age. PLoS ONE.

[4] Koch CM, Wagner W (2011). Epigenetic-aging-signature to determine age in different tissues. Aging (Albany NY).

[5] Weidner CI, Lin Q, Koch CM, Eisele L, Beier F, Ziegler P (2014). Aging of blood can be tracked by DNA methylation changes at just three CpG sites. Genome Biology.

[6] Manev H, Dzitoyeva S, Chen H (2012). Mitochondrial DNA: a blind spot in neuroepigenetics. Biomolecular concepts.

[7] Hong EE, Okitsu CY, Smith AD, Hsieh C-L (2013). Regionally specific and genome-wide analyses conclusively demonstrate the absence of CpG methylation in human mitochondrial DNA. Molecular and cellular biology.

[8] Maekawa M, Taniguchi T, Higashi H, Sugimura H, Sugano K, Kanno T (2004). Methylation of mitochondrial DNA is not a useful marker for cancer detection. Clinical chemistry.

[9] Stewart SA, Weinberg RA (2002). Senescence: does it all happen at the ends?. Oncogene.

[10] Stewart SA, Weinberg RA (2006). Telomeres: cancer to human aging. Annu Rev Cell Dev Biol.

[11] Shock LS, Thakkar PV, Peterson EJ, Moran RG, Taylor SM (2011). DNA methyltransferase 1, cytosine methylation, and cytosine hydroxymethylation in mammalian mitochondria. Proceedings of the National Academy of Sciences.

[12] Chinnery PF, Elliott HR, Hudson G, Samuels DC, Relton CL (2012). Epigenetics, epidemiology and mitochondrial DNA diseases. International journal of epidemiology.

[13] Iacobazzi V, Castegna A, Infantino V, Andria G (2013). Mitochondrial DNA methylation as a next-generation biomarker and diagnostic tool. Molecular Genetics and Metabolism.

[14] Bellizzi D, D'Aquila P, Scafone T, Giordano M, Riso V, Riccio A (2013). The Control Region of Mitochondrial DNA Shows an Unusual CpG and Non-CpG Methylation Pattern. DNA Research.

[15] Byun H-M, Panni T, Motta V, Hou L, Nordio F, Apostoli P (2013). Effects of airborne pollutants on mitochondrial DNA methylation. Part Fibre Toxicol.

[16] O'Sullivan M, Rutland P, Lucas D, Ashton E, Hendricks S, Rahman S (2014). Mitochondrial m. 1584A 12S m62A rRNA methylation in families with m. 1555A> G associated hearing loss. Human molecular genetics.

[17] Metodiev MD, Lesko N, Park CB, Cámara Y, Shi Y, Wibom R (2009). Methylation of 12S rRNA is necessary for in vivo stability of the small subunit of the mammalian mitochondrial ribosome. Cell metabolism.

[18] Rebelo AP, Williams SL, Moraes CT (2009). In vivo methylation of mtDNA reveals the dynamics of protein-mtDNA interactions. Nucleic acids research.

[19] Feng S, Xiong L, Ji Z, Cheng W, Yang H (2012). Correlation between increased ND2 expression and demethylated displacement loop of mtDNA in colorectal cancer. Molecular medicine reports.

[20] Pirola CJ, Gianotti TF, Burgueño AL, Rey-Funes M, Loidl CF, Mallardi P (2012). Epigenetic modification of liver mitochondrial DNA is associated with histological severity of nonalcoholic fatty liver disease. Gut.

[21] Komaki H, Akanuma J, Iwata H, Takahashi T, Mashima Y, Nonaka I (2003). A novel mtDNA C11777A mutation in Leigh syndrome. Mitochondrion.

[22] Harding A, Holt I, Sweeney M, Brockington M, Davis M (1992). Prenatal diagnosis of mitochondrial DNA8993 T----G disease. American journal of human genetics.

[23] Holt I, Harding A, Petty R, Morgan-Hughes J (1990). A new mitochondrial disease associated with mitochondrial DNA heteroplasmy. American journal of human genetics.

[24] Butler JM (2005). Forensic DNA typing biology, technology, and genetics of STR markers.

[25] RESEARCH Z (2014). EZ DNA Methylation-Direct™ Kit. Zymo Research.

[26] Ghosh S, Sengupta S, Scaria V (2014). Comparative analysis of human mitochondrial methylomes shows distinct patterns of epigenetic regulation in mitochondria. Mitochondrion.

[27] Xu C, Qu H, Wang G, Xie B, Shi Y, Yang Y (2014). A novel strategy for forensic age prediction by DNA methylation and support vector regression model. Scientific reports.

[28] Giordano M, Cristiani C, Crocco P, D'Aquila P, De Rango F, Pisani F (2012). Methylation of the human mitochondrial 12S rRNA gene is correlated with aging. 12th International FISV Congress, Rome, Italy.

[29] Park J-L, Kwon O-H, Kim JH, Yoo H-S, Lee H-C, Woo K-M (2014). Identification of body fluid-specific DNA methylation markers for use in forensic science. Forensic Science International: Genetics.

[30] Antunes JP, Madi T, Balamurugan K, Bombardi R, Duncan G, McCord B (2014). DNA methylation markers as a powerful technique to discriminate body fluids present in crime scenes.

