# Supplementary material for: Quantification of global mitochondrial DNA methylation levels and inverse correlation with age at two CpG sites
Source: Aging (Albany NY). 2016 Feb 17;8(4):636–41. doi: 10.18632/aging.100892 (PMC4925819; doi:10.18632/aging.100892)
Supplement: Supplementary file 1 [file aging-08-636-s001.pdf]

24. Butler JM. Forensic DNA typing biology, technology, and genetics of STR markers. Amsterdam; Boston: Elsevier Academic Press. 2005.

25. RESEARCH Z. EZ DNA Methylation-Direct™ Kit. Zymo Research. 2014.

26. Ghosh S, Sengupta S, Scaria V. Comparative analysis of human mitochondrial methylomes shows distinct patterns of epigenetic regulation in mitochondria. Mitochondrion. 2014; 18:58-62.

27. Xu C, Qu H, Wang G, Xie B, Shi Y, Yang Y, et al. A novel strategy for forensic age prediction by DNA methylation and support vector regression model. Scientific reports. 2014; 5:17788.

28. Giordano M, Cristiani C, Crocco P, D'Aquila P, De Rango F, Pisani F, et al. Methylation of the human mitochondrial 12S rRNA gene is correlated with aging. 12th International FISV Congress, Rome, Italy. 2012; 9.5.

29. Park J-L, Kwon O-H, Kim JH, Yoo H-S, Lee H-C, Woo K-M, et al. Identification of body fluid-specific DNA methylation markers for use in forensic science. Forensic Science International: Genetics. 2014; 13:147-53.

30. Antunes JP, Madi T, Balamurugan K, Bombardi R, Duncan G, McCord B. DNA methylation markers as a powerful technique to discriminate body fluids present in crime scenes. Proceedings of the 24th International Symposium on Human Identification. 2014.

SUPPLEMENTARY DATA

Please browse the Full text version of this manuscript to see the Supplementary material.

Supplementary 1. The regions of interest (ROI) with their sequences and primer sequences (forward and reverse). The single strand sequences of the ROI after bisulphite sodium conversion with the primers sequence (forward and reverse).

Supplementary 2. Reference Sequence: The Mitochondrial sequence (hg38:2013) shows all the 345 CG (underlined and bold **CG**) and 54 assessed sites indicated by red text with underlined and bold **CG**.
