# Supplementary material for: Quantification of global mitochondrial DNA methylation levels and inverse correlation with age at two CpG sites
Source: Aging (Albany NY). 2016 Feb 17;8(4):636–41. doi: 10.18632/aging.100892 (PMC4925819; doi:10.18632/aging.100892)
Supplement: Supplementary file 2 [file aging-08-636-s002.pdf]

Supplementary 1: Reference Sequence: The Mitochondrial sequence (hg38:2013) shows all the 345 CG (underline and bold **CG**) and 53 red colour with underline and bold **CG**.

1 GATCACAGGTCTATCACCCTATTAACCACTCA**CG**GGAGCTCTCCATGCATTTGGTATTTT
61 **CG**TCTGGGGGGTATGCA**CGCG**ATAGCATTG**CG**AGA**CG**CTGGAGC**CG**GAGCACCCTATGT**C**
121 **G**CAGTATCTGTCTTTGATTCCTGCCTCATCCTATTATTTAT**CG**CACCTA**CG**TTCAATATT
181 ACAGG**CG**AACATACTTACTAAAGTGTGTTAATTAATTAATGCTTGTAGGACATAATAATA
241 ACAATTGAATGTCTGCACAGCCACTTTCCACACAGACATCATAACAAAAAATTTCCACCA
301 AACCCCCCCTCCCC**CG**CTTCTGGCCACAGCACTTAAACACATCTCTGCCAAACCCCAAAA
361 ACAAAGAACCCTAACACCAGCCTAACCAGATTTCAAATTTTATCTTTTGG**CG**GTATGCAC
421 TTTTAACAGTCACCCCCCAACTAACACATTATTTTCCCCTCCCACTCCCATACTACTAAT
481 CTCATCAATACAACCCC**CG**CCCATCCTACCCAGCACACACACAC**CG**CTGCTAACCCCATA
541 CCC**CG**AACCAACCAAACCCCAAAGACACCCCCCACAGTTTATGTAGCTTACCTCCTCAAA
601 GCAATACACTGAAAATGTTTAGA**CG**GGCTCACATCACCCCATAAACAAATAGGTTTGGTC
661 CTAGCCTTTCTATTAGCTCTTAGTAAGATTACACATGCAAGCATCCC**CG**TTCCAGTGAGT
721 TCACCCTCTAAATCACCA**CG**ATCAAAAGGAACAAGCATCAAGCA**CG**CAGCAATGCAGCTC
781 AAAA**CG**CTTAGCCTAGCCACACCCCCA**CG**GGAAACAGCAGTGATTAACCTTTAGCAATAA
841 A**CG**AAAGTTTAACTAAGCTATACTAACCCCAGGGTTGGTCAATTT**CG**TGCCAGCCAC**CGC**
901 **G**GTCACA**CG**ATTAACCCAAGTCAATAGAAGC**CG**G**CG**TAAAGAGTGTTTTAGATCACCCCC
961 TCCCCAATAAAGCTAAAACTCACCTGAGTTGTAAAAAACTCCAGTTGACACAAAATAGAC
1021 TA**CG**AAAGTGGCTTTAACATATCTGAACACACAATAGCTAAGACCCAAACTGGGATTAGA
1081 TACCCCACTATGCTTAGCCCTAAACCTCAACAGTTAAATCAACAAAACTGCT**CG**CCAGAA
1141 CACTA**CG**AGCACAGCTTAAAACTCAAAGGACCTGG**CG**GTGCTTCATATCCCTCTAGAGGA
1201 GCCTGTTCTGTAAT**CG**ATAAACCC**CG**ATCAACCTCACCACCTCTTGCTCAGCCTATATAC
1261 **CG**CCATCTTCAGCAAACCCTGATGAAGGCTACAAAGTAAG**CG**CAAGTACCCA**CG**TAAAGA
1321 **CG**TTAGGTCAAGGTGTAGCCCATGAGGTGGCAAGAAATGGGCTACATTTTCTACCCCAGA
1381 AAACTA**CG**ATAGCCCTTATGAAACTTAAGGGT**CG**AAGGTGGATTTAGCAGTAAACTAAGA
1441 GTAGAGTGCTTAGTTGAACAGGGCCCTGAAG**CGCG**TACACAC**CG**CC**CG**TCACCCTCCTCA
1501 AGTATACTTCAAAGGACATTTAACTAAAACCCCTA**CG**CATTTATATAGAGGAGACAAGT**C**
1561 **G**TAACATGGTAAGTGTACTGGAAAGTGCACTTGGA**CG**AACCAGAGTGTAGCTTAACACAA
1621 AGCACCCAACTTACACTTAGGAGATTTCAACTTAACTTGAC**CG**CTCTGAGCTAAACCTAG
1681 CCCCAAACCCACTCCACCTTACTACCAGACAACCTTAGCCAAACCATTTACCCAAATAAA
1741 GTATAGG**CG**ATAGAAATTGAAACCTGG**CG**CAATAGATATAGTAC**CG**CAAGGGAAAGATGA
1801 AAAATTATAACCAAGCATAATATAGCAAGGACTAACCCCTATACCTTCTGCATAATGAAT
1861 TAACTAGAAATAACTTTGCAAGGAGAGCCAAAGCTAAGACCCC**CG**AAACCAGA**CG**AGCTA
1921 CCTAAGAACAGCTAAAAGAGCACACC**CG**TCTATGTAGCAAAATAGTGGGAAGATTTATAG
1981 GTAGAGG**CG**ACAAACCTAC**CG**AGCCTGGTGATAGCTGGTTGTCCAAGATAGAATCTTAGT
2041 TCAACTTTAAATTTGCCCACAGAACCCTCTAAATCCCCTTGTAAATTTAACTGTTAGTCC
2101 CAAAGAGGAACAGCTCTTTGGACACTAGGAAAAAACCTTGTAGAGAGAGTAAAAAATTTA
2161 CACCCATAGTAGGCCTAAAAGCAGCCACCAATTAAGAAAG**CG**TTCAAGCTCAACACCCAC
2221 TACCTAAAAAATCCCAAACATATAACTGAACTCCTCACACCCAATTGGACCAATCTATCA
2281 CCCTATAGAAGAACTAATGTTAGTATAAGTAACATGAAAACATTCTCCTC**CG**CATAAGCC
2341 TG**CG**TCAGATTAAAACACTGAACTGACAATTAACAGCCCAATATCTACAATCAACCAACA
2401 AGTCATTATTACCCTCACTGTCAACCCAACACAGGCATGCTCATAAGGAAAGGTTAAAAA
2461 AAGTAAAAGGAACT**CG**GCAAATCTTACCC**CG**CCTGTTTACCAAAAACATCACCTCTAGCA
2521 TCACCAGTATTAGAGGCAC**CG**CCTGCCCAGTGACACATGTTTAA**CG**GC**CGCG**GTACCCTA
2581 AC**CG**TGCAAAGGTAGCATAATCACTTGTTCCTTAAATAGGGACCTGTATGAATGGCTCCA
2641 **CG**AGGGTTCAGCTGTCTCTTACTTTTAACCAGTGAAATTGACCTGCC**CG**TGAAGAGG**CG**G
2701 GCATAACACAGCAAGA**CG**AGAAGACCCTATGGAGCTTTAATTTATTAATGCAAACAGTAC
2761 CTAACAAACCCACAGGTCCTAAACTACCAAACCTGCATTAAAAATTT**CG**GTTGGGG**CG**AC
2821 CT**CG**GAGCAGAACCCAACCTC**CG**AGCAGTACATGCTAAGACTTCACCAGTCAAAG**CG**AAC
2881 TACTATACTCAATTGATCCAATAACTTGACCAA**CG**GAACAAGTTACCCTAGGGATAACAG
2941 **CG**CAATCCTATTCTAGAGTCCATATCAACAATAGGGTTTA**CG**ACCT**CG**ATGTTGGATCAG
3001 GACATCC**CG**ATGGTGCAGC**CG**CTATTAAAGGTT**CG**TTTGTTCAA**CG**ATTAAAGTCCTA**CG**
3061 TGATCTGAGTTCAGAC**CG**GAGTAATCCAGGT**CG**GTTTCTATCTACNTTCAAATTCCTCCC
3121 TGTA**CG**AAAGGACAAGAGAAATAAGGCCTACTTCACAAAG**CG**CCTTCCCC**CG**TAAATGAT
3181 ATCATCTCAACTTAGTATTATACCCACACCCACCCAAGAACAGGGTTTGTTAAGATGGCA
3241 GAGCC**CG**GTAAT**CG**CATAAAACTTAAAACTTTACAGTCAGAGGTTCAATTCCTCTTCTTA
3301 ACAACATACCCATGGCCAACCTCCTACTCCTCATTGTACCCATTCTAAT**CG**CAATGGCAT

3361 TCCTAATGCTTACCGAACGAAAAATTCTAGGCTATATACAACTACGCAAAGGCCCCAACG
3421 TTGTAGGCCCCTACGGGCTACTACAACCCTTCGCTGACGCCATAAAACTCTTCACCAAAG
3481 AGCCCCTAAAACCCGCCACATCTACCATCACCCTCTACATCACCGCCCCGACCTTAGCTC
3541 TCACCATCGCTCTTCTACTATGAACCCCCCTCCCCATACCCAACCCCCTGGTCAACCTCA
3601 ACCTAGGCCTCCTATTTATTCTAGCCACCTCTAGCCTAGCCGTTTACTCAATCCTCTGAT
3661 CAGGGTGAGCATCAAACTCAAACTACGCCCTGATCGGCGCACTGCGAGCAGTAGCCCAAA
3721 CAATCTCATATGAAGTCACCCTAGCCATCATTCTACTATCAACATTACTAATAAGTGGCT
3781 CCTTTAACCTCTCCACCCTTATCACAACACAAGAACACCTCTGATTACTCCTGCCATCAT
3841 GACCCTTGGCCATAATATGATTTATCTCCACACTAGCAGAGACCAACCGAACCCCCTTCG
3901 ACCTTGCCGAAGGGGAGTCCGAACTAGTCTCAGGCTTCAACATCGAATACGCCGCAGGCC
3961 CCTTCGCCCTATTCTTCATAGCCGAATACACAAACATTATTATAATAAACACCCTCACCA
4021 CTACAATCTTCCTAGGAACAACATATGACGCACTCTCCCCTGAACTCTACACAACATATT
4081 TTGTCACCAAGACCCTACTTCTAACCTCCCTGTTCTTATGAATTCGAACAGCATACCCCC
4141 GATTCCGCTACGACCAACTCATACACCTCCTATGAAAAAACTTCCTACCACTCACCCTAG
4201 CATTACTTATATGATATGTCTCCATACCCATTACAATCTCCAGCATTCCCCCTCAAACCT
4261 AAGAAATATGTCTGATAAAAGAGTTACTTTGATAGAGTAAATAATAGGAGCTTAAACCCC
4321 CTTATTTCTAGGACTATGAGAATCGAACCCATCCCTGAGAATCCAAAATTCTCCGTGCCA
4381 CCTATCACACCCCATCCTAAAGTAAGGTCAGCTAAATAAGCTATCGGGCCCATACCCCGA
4441 AAATGTTGGTTATACCCTTCCCGTACTAATTAATCCCCTGGCCCAACCCGTCATCTACTC
4501 TACCATCTTTGCAGGCACACTCATCACAGCGCTAAGCTCGCACTGATTTTTTACCTGAGT
4561 AGGCCTAGAAATAAACATGCTAGCTTTTATTCCAGTTCTAACCAAAAAAATAAACCCTCG
4621 TTCCACAGAAGCTGCCATCAAGTATTTCCTCACGCAAGCAACCGCATCCATAATCCTTCT
4681 AATAGCTATCCTCTTCAACAATATACTCTCCGGACAATGAACCATAACCAATACTACCAA
4741 TCAATACTCATCATTAATAATCATAATAGCTATAGCAATAAAACTAGGAATAGCCCCCTT
4801 TCACTTCTGAGTCCCAGAGGTTACCCAAGGCACCCCTCTGACATCCGGCCTGCTTCTTCT
4861 CACATGACAAAAACTAGCCCCCATCTCAATCATATACCAAATCTCTCCCTCACTAAACGT
4921 AAGCCTTCTCCTCACTCTCTCAATCTTATCCATCATAGCAGGCAGTTGAGGTGGATTAAA
4981 CCAAACCCAGCTACGCAAAATCTTAGCATACTCCTCAATTACCCACATAGGATGAATAAT
5041 AGCAGTTCTACCGTACAACCCTAACATAACCATTCTTAATTTAACTATTTATATTATCCT
5101 AACTACTACCGCATTCCTACTACTCAACTTAAACTCCAGCACCACGACCCTACTACTATC
5161 TCGCACCTGAAACAAGCTAACATGACTAACACCCTTAATTCCATCCACCCTCCTCTCCCT
5221 AGGAGGCCTGCCCCCGCTAACCGGCTTTTTGCCCAAATGGGCCATTATCGAAGAATTCAC
5281 AAAAAACAATAGCCTCATCATCCCCACCATCATAGCCACCATCACCCTCCTTAACCTCTA
5341 CTTCTACCTACGCCTAATCTACTCCACCTCAATCACACTACTCCCCATATCTAACAACGT
5401 AAAAATAAAATGACAGTTTGAACATACAAAACCCACCCCATTCCTCCCCACACTCATCGC
5461 CCTTACCACGCTACTCCTACCTATCTCCCCTTTTATACTAATAATCTTATAGAAATTTAG
5521 GTTAAATACAGACCAAGAGCCTTCAAAGCCCTCAGTAAGTTGCAATACTTAATTTCTGTA
5581 ACAGCTAAGGACTGCAAAACCCCACTCTGCATCAACTGAACGCAAATCAGCCACTTTAAT
5641 TAAGCTAAGCCCTTACTAGACCAATGGGACTTAAACCCACAAACACTTAGTTAACAGCTA
5701 AGCACCCTAATCAACTGGCTTCAATCTACTTCTCCCGCCGCCGGGAAAAAAGGCGGGAGA
5761 AGCCCCGGCAGGTTTGAAGCTGCTTCTTCGAATTTGCAATTCAATATGAAAATCACCTCG
5821 GAGCTGGTAAAAAGAGGCCTAACCCCTGTCTTTAGATTTACAGTCCAATGCTTCACTCAG
5881 CCATTTTACCTCACCCCCACTGATGTTCGCCGACCGTTGACTATTCTCTACAAACCACAA
5941 AGACATTGGAACACTATACCTATTATTCGGCGCATGAGCTGGAGTCCTAGGCACAGCTCT
6001 AAGCCTCCTTATTCGAGCCGAGCTGGGCCAGCCAGGCAACCTTCTAGGTAACGACCACAT
6061 CTACAACGTTATCGTCACAGCCCATGCATTTGTAATAATCTTCTTCATAGTAATACCCAT
6121 CATAATCGGAGGCTTTGGCAACTGACTAGTTCCCCTAATAATCGGTGCCCCCGATATGGC
6181 GTTTCCCCGCATAAACAACATAAGCTTCTGACTCTTACCTCCCTCTCTCCTACTCCTGCT
6241 CGCATCTGCTATAGTGGAGGCCGGAGCAGGAACAGGTTGAACAGTCTACCCTCCCTTAGC
6301 AGGGAACTACTCCCACCCTGGAGCCTCCGTAGACCTAACCATCTTCTCCTTACACCTAGC
6361 AGGTGTCTCCTCTATCTTAGGGGCCATCAATTTCATCACAACAATTATCAATATAAAACC
6421 CCCTGCCATAACCCAATACCAAACGCCCCTCTTCGTCTGATCCGTCCTAATCACAGCAGT
6481 CCTACTTCTCCTATCTCTCCCAGTCCTAGCTGCTGGCATCACTATACTACTAACAGACCG
6541 CAACCTCAACACCACCTTCTTCGACCCCGCCGGAGGAGGAGACCCCATTCTATACCAACA
6601 CCTATTCTGATTTTTCGGTCACCCTGAAGTTTATATTCTTATCCTACCAGGCTTCGGAAT
6661 AATCTCCCATATTGTAACTTACTACTCCGGAAAAAAAGAACCATTTGGATACATAGGTAT
6721 GGTCTGAGCTATGATATCAATTGGCTTCCTAGGGTTTATCGTGTGAGCACACCATATATT
6781 TACAGTAGGAATAGACGTAGACACACGAGCATATTTCACCTCCGCTACCATAATCATCGC
6841 TATCCCCACCGGCGTCAAAGTATTTAGCTGACTCGCCACACTCCACGGAAGCAATATGAA
6901 ATGATCTGCTGCAGTGCTCTGAGCCCTAGGATTCATCTTTCTTTTCACCGTAGGTGGCCT
6961 GACTGGCATTGTATTAGCAAACTCATCACTAGACATCGTACTACACGACACGTACTACGT

7021 TGTAGCCCACTTCCACTATGTCCTATCAATAGGAGCTGTATTTGCCATCATAGGAGGCTT
7081 CATTCACTGATTTCCCCTATTCTCAGGCTACACCCTAGACCAAACCTACGCCAAAATCCA
7141 TTTCACTATCATATTCATCGGCGTAAATCTAACTTTCTTCCCACAACACTTTCTCGGCCT
7201 ATCCGGAATGCCCCGACGTTACTCGGACTACCCCGATGCATACACCACATGAAACATCCT
7261 ATCATCTGTAGGCTCATTCATTTCTCTAACAGCAGTAATATTAATAATTTTCATGATTTG
7321 AGAAGCCTTCGCTTCGAAGCGAAAAGTCCTAATAGTAGAAGAACCCTCCATAAACCTGGA
7381 GTGACTATATGGATGCCCCCCACCCTACCACACATTCGAAGAACCCGTATACATAAAATC
7441 TAGACAAAAAAGGAAGGAATCGAACCCCCCAAAGCTGGTTTCAAGCCAACCCCATGGCCT
7501 CCATGACTTTTTCAAAAAGGTATTAGAAAAACCATTTCATAACTTTGTCAAAGTTAAATT
7561 ATAGGCTAAATCCTATATATCTTAATGGCACATGCAGCGCAAGTAGGTCTACAAGACGCT
7621 ACTTCCCCTATCATAGAAGAGCTTATCACCTTTCATGATCACGCCCTCATAATCATTTTC
7681 CTTATCTGCTTCCTAGTCCTGTATGCCCTTTTCCTAACACTCACAACAAAACTAACTAAT
7741 ACTAACATCTCAGACGCTCAGGAAATAGAAACCGTCTGAACTATCCTGCCCGCCATCATC
7801 CTAGTCCTCATCGCCCTCCCATCCCTACGCATCCTTTACATAACAGACGAGGTCAACGAT
7861 CCCTCCCTTACCATCAAATCAATTGGCCACCAATGGTACTGAACCTACGAGTACACCGAC
7921 TACGGCGGACTAATCTTCAACTCCTACATACTTCCCCCATTATTCCTAGAACCAGGCGAC
7981 CTGCGACTCCTTGACGTTGACAATCGAGTAGTACTCCCGATTGAAGCCCCCATTCGTATA
8041 ATAATTACATCACAAGACGTCTTGCACTCATGAGCTGTCCCCACATTAGGCTTAAAAACA
8101 GATGCAATTCCCGGACGTCTAAACCAAACCACTTTCACCGCTACACGACCGGGGGTATAC
8161 TACGGTCAATGCTCTGAAATCTGTGGAGCAAACCACAGTTTCATGCCCATCGTCCTAGAA
8221 TTAATTCCCCTAAAAATCTTTGAAATAGGGCCCGTATTTACCCTATAGCACCCCCTCTAC
8281 CCCCTCTAGAGCCCACTGTAAAGCTAACTTAGCATTAACCTTTTAAGTTAAAGATTAAGA
8341 GAACCAACACCTCTTTACAGTGAAATGCCCCAACTAAATACTACCGTATGGCCCACCATA
8401 ATTACCCCCATACTCCTTACACTATTCCTCATCACCCAACTAAAAATATTAAACACAAAC
8461 TACCACCTACCTCCCTCACCAAAGCCCATAAAAATAAAAAATTATAACAAACCCTGAGAA
8521 CCAAAATGAACGAAAATCTGTTCGCTTCATTCATTGCCCCCACAATCCTAGGCCTACCCG
8581 CCGCAGTACTGATCATTCTATTTCCCCCTCTATTGATCCCCACCTCCAAATATCTCATCA
8641 ACAACCGACTAATCACCACCCAACAATGACTAATCAAACTAACCTCAAAACAAATGATAA
8701 CCATACACAACACTAAAGGACGAACCTGATCTCTTATACTAGTATCCTTAATCATTTTTA
8761 TTGCCACAACTAACCTCCTCGGACTCCTGCCTCACTCATTTACACCAACCACCCAACTAT
8821 CTATAAACCTAGCCATGGCCATCCCCTTATGAGCGGGCACAGTGATTATAGGCTTTCGCT
8881 CTAAGATTAAAAATGCCCTAGCCCACTTCTTACCACAAGGCACACCTACACCCCTTATCC
8941 CCATACTAGTTATTATCGAAACCATCAGCCTACTCATTCAACCAATAGCCCTGGCCGTAC
9001 GCCTAACCGCTAACATTACTGCAGGCCACCTACTCATGCACCTAATTGGAAGCGCCACCC
9061 TAGCAATATCAACCATTAACCTTCCCTCTACACTTATCATCTTCACAATTCTAATTCTAC
9121 TGACTATCCTAGAAATCGCTGTCGCCTTAATCCAAGCCTACGTTTTCACACTTCTAGTAA
9181 AGCCTCTACCTGCACGACAACACATAATGACCCACCAATCACATGCCTATCATATAGTAA
9241 ACCCAGCCCATGACCCCTAACAGGGGCCCTCTCAGCCCTCCTAATGACCTCCGGCCTAGC
9301 CATGTGATTTCACTTCCACTCCATAACGCTCCTCATACTAGGCCTACTAACCAACACACT
9361 AACCATATACCAATGATGGCGCGATGTAACACGAGAAAGCACATACCAAGGCCACCACAC
9421 ACCACCTGTCCAAAAAGGCCTTCGATACGGGATAATCCTATTTATTACCTCAGAAGTTTT
9481 TTTCTTCGCAGGATTTTTCTGAGCCTTTTACCACTCCAGCCTAGCCCCTACCCCCCAATT
9541 AGGAGGGCACTGGCCCCCAACAGGCATCACCCCGCTAAATCCCCTAGAAGTCCCACTCCT
9601 AAACACATCCGTATTACTCGCATCAGGAGTATCAATCACCTGAGCTCACCATAGTCTAAT
9661 AGAAAACAACCGAAACCAAATAATTCAAGCACTGCTTATTACAATTTTACTGGGTCTCTA
9721 TTTTACCCTCCTACAAGCCTCAGAGTACTTCGAGTCTCCCTTCACCATTTCCGACGGCAT
9781 TCTACGGCTCAACATTTTTTGTAGCCACAGGCTTCCACGGACTTCACGTCATTATTGGCT
9841 AACTTTCCTCACTATCTGCTTCATCCGCCAACTAATATTTCACTTTACATCCAAACATCA
9901 CTTTGGCTTCGAAGCCGCCGCCTGATACTGGCATTTTGTAGATGTGGTTTGACTATTTCT
9961 GTATGTCTCCATCTATTGATGAGGGTCTTACTCTTTTAGTATAAATAGTACCGTTAACTT
10021 CCAATTAACTAGTTTTGACAACATTCAAAAAAGAGTAATAAACTTCGCCTTAATTTTAAT
10081 AATCAACACCCTCCTAGCCTTACTACTAATAATTATTACATTTTGACTACCACAACTCAA
10141 CGGCTACATAGAAAAATCCACCCCTTACGAGTGCGGCTTCGACCCTATATCCCCCGCCCG
10201 CGTCCCTTTCTCCATAAAATTCTTCTTAGTAGCTATTACCTTCTTATTATTTGATCTAGA
10261 AATTGCCCTCCTTTTACCCCTACCATGAGCCCTACAAACAACTAACCTGCCACTAATAGT
10321 TATGTCATCCCTCTTATTAATCATCATCCTAGCCCTAAGTCTGGCCTATGAGTGACTACA
10381 AAAAGGATTAGACTGAACCGAATTGGTATATAGTTTAAACAAAACGAATGATTTCGACTC
10441 ATTAAATTATGATAATCATATTTACCAAATGCCCCTCATTTACATAAATATTATACTAGC
10501 ATTTACCATCTCACTTCTAGGAATACTAGTATATCGCTCACACCTCATATCCTCCCTACT
10561 ATGCCTAGAAGGAATAATACTATCGCTGTTCATTATAGCTACTCTCATAACCCTCAACAC
10621 CCACTCCCTCTTAGCCAATATTGTGCCTATTGCCATACTAGTCTTTGCCGCCTGCGAAGC

10681 AGCGGTGGGCCTAGCCCTACTAGTCTCAATCTCCAACACATATGGCCTAGACTACGTACA
10741 TAACCTAAACCTACTCCAATGCTAAAACTAATCGTCCCAACAATTATATTACTACCACTG
10801 ACATGACTTTCCAAAAAACACATAATTTGAATCAACACAACCACCCACAGCCTAATTATT
10861 AGCATCATCCCTCTACTATTTTTTAACCAAATCAACAACAACCTATTTAGCTGTTCCCCA
10921 ACCTTTTCCTCCGACCCCCTAACAACCCCCCTCCTAATACTAACTACCTGACTCCTACCC
10981 CTCACAATCATGGCAAGCCAACGCCACTTATCCAGTGAACCACTATCACGAAAAAAACTC
11041 TACCTCTCTATACTAATCTCCCTACAAATCTCCTTAATTATAACATTCACAGCCACAGAA
11101 CTAATCATATTTTATATCTTCTTCGAAACCACACTTATCCCCACCTTGGCTATCATCACC
11161 CGATGAGGCAACCAGCCAGAACGCCTGAACGCAGGCACATACTTCCTATTCTACACCCTA
11221 GTAGGCTCCCTTCCCCTACTCATCGCACTAATTTACACTCACAACACCCTAGGCTCACTA
11281 AACATTCTACTACTCACTCTCACTGCCCAAGAACTATCAAACTCCTGAGCCAACAACTTA
11341 ATATGACTAGCTTACACAATAGCTTTTATAGTAAAGATACCTCTTTACGGACTCCACTTA
11401 TGACTCCCTAAAGCCCATGTCGAAGCCCCCATCGCTGGGTCAATAGTACTTGCCGCAGTA
11461 CTCTTAAAACTAGGCGGCTATGGTATAATACGCCTCACACTCATTCTCAACCCCCTGACA
11521 AAACACATAGCCTACCCCTTCCTTGTACTATCCCTATGAGGCATAATTATAACAAGCTCC
11581 ATCTGCCTACGACAAACAGACCTAAAATCGCTCATTGCATACTCTTCAATCAGCCACATA
11641 GCCCTCGTAGTAACAGCCATTCTCATCCAAACCCCCTGAAGCTTCACCGGCGCAGTCATT
11701 CTCATAATCGCCCACGGGCTTACATCCTCATTACTATTCTGCCTAGCAAACTCAAACTAC
11761 GAACGCACTCACAGTCGCATCATAATCCTCTCTCAAGGACTTCAAACTCTACTCCCACTA
11821 ATAGCTTTTTGATGACTTCTAGCAAGCCTCGCTAACCTCGCCTTACCCCCCACTATTAAC
11881 CTACTGGGAGAACTCTCTGTGCTAGTAACCACGTTCTCCTGATCAAATATCACTCTCCTA
11941 CTTACAGGACTCAACATACTAGTCACAGCCCTATACTCCCTCTACATATTTACCACAACA
12001 CAATGGGGCTCACTCACCCACCACATTAACAACATAAAACCCTCATTCACACGAGAAAAC
12061 ACCCTCATGTTCATACACCTATCCCCCATTCTCCTCCTATCCCTCAACCCCGACATCATT
12121 ACCGGGTTTTCCTCTTGTAAATATAGTTTAACCAAAACATCAGATTGTGAATCTGACAAC
12181 AGAGGCTTACGACCCCTTATTTACCGAGAAAGCTCACAAGAACTGCTAACTCATGCCCCC
12241 ATGTCTAACAACATGGCTTTCTCAACTTTTAAAGGATAACAGCTATCCATTGGTCTTAGG
12301 CCCCAAAAATTTTGGTGCAACTCCAAATAAAAGTAATAACCATGCACACTACTATAACCA
12361 CCCTAACCCTGACTTCCCTAATTCCCCCCATCCTTACCACCCTCGTTAACCCTAACAAAA
12421 AAAACTCATACCCCCATTATGTAAAATCCATTGTCGCATCCACCTTTATTATCAGTCTCT
12481 TCCCCACAACAATATTCATGTGCCTAGACCAAGAAGTTATTATCTCGAACTGACACTGAG
12541 CCACAACCCAAACAACCCAGCTCTCCCTAAGCTTCAAACTAGACTACTTCTCCATAATAT
12601 TCATCCCTGTAGCATTGTTCGTTACATGGTCCATCATAGAATTCTCACTGTGATATATAA
12661 ACTCAGACCCAAACATTAATCAGTTCTTCAAATATCTACTCATCTTCCTAATTACCATAC
12721 TAATCTTAGTTACCGCTAACAACCTATTCCAACTGTTCATCGGCTGAGAGGGCGTAGGAA
12781 TTATATCCTTCTTGCTCATCAGTTGATGATACGCCCGAGCAGATGCCAACACAGCAGCCA
12841 TTCAAGCAATCCTATACAACCGTATCGGCGATATCGGTTTCATCCTCGCCTTAGCATGAT
12901 TTTATCCTACACTCCAACTCATGAGACCCACAACAAATAGCCCTTCTAAACGCTAATCCA
12961 GCCTCACCCCACTACTAGGCCTCCTCCTAGCAGCAGCAGGCAAATCAGCCCAATTAGGTC
13021 TCCACCCCTGACTCCCCTCAGCCATAGAAGGCCCCACCCCAGTCTCAGCCCTACTCCACT
13081 CAAGCACTATAGTTGTAGCAGGAATCTTCTTACTCATCCGCTTCCACCCCCTAGCAGAAA
13141 ATAGCCCACTAATCCAAACTCTAACACTATGCTTAGGCGCTATCACCACTCTGTTCGCAG
13201 CAGTCTGCGCCCTTACACAAAATGACATCAAAAAAATCGTAGCCTTCTCCACTTCAAGTC
13261 AACTAGGACTCATAATAGTTACAATCGGCATCAACCAACCACACCTAGCATTCCTGCACA
13321 TCTGTACCCACGCCTTCTTCAAAGCCATACTATTTATGTGCTCCGGGTCCATCATCCACA
13381 ACCTTAACAATGAACAAGATATTCGAAAAATAGGAGGACTACTCAAAACCATACCTCTCA
13441 CTTCAACCTCCCTCACCATTGGCAGCCTAGCATTAGCAGGAATACCTTTCCTCACAGGTT
13501 TCTACTCCAAAGACCACATCATCGAAACCGCAAACATATCATACACAAACGCCTGAGCCC
13561 TATCTATTACTCTCATCGCTACCTCCCTGACAAGCGCCTATAGCACTCGAATAATTCTTC
13621 TCACCCTAACAGGTCAACCTCGCTTCCCCACCCTTACTAACATTAACGAAAATAACCCCA
13681 CCCTACTAAACCCCATTAAACGCCTGGCAGCCGGAAGCCTATTCGCAGGATTTCTCATTA
13741 CTAACAACATTTCCCCCGCATCCCCCTTCCAAACAACAATCCCCCTCTACCTAAAACTCA
13801 CAGCCCTCGCTGTCACTTTCCTAGGACTTCTAACAGCCCTAGACCTCAACTACCTAACCA
13861 ACAAACTTAAAATAAAATCCCCACTATGCACATTTTATTTCTCCAACATACTCGGATTCT
13921 ACCCTAGCATCACACACCGCACAATCCCCTATCTAGGCCTTCTTACGAGCCAAAACCTGC
13981 CCCTACTCCTCCTAGACCTAACCTGACTAGAAAAGCTATTACCTAAAACAATTTCACAGC
14041 ACCAAATCTCCACCTCCATCATCACCTCAACCCAAAAAGGCATAATTAAACTTTACTTCC
14101 TCTCTTTCTTCTTCCCACTCATCCTAACCCTACTCCTAATCACATAACCTATTCCCCCGA
14161 GCAATCTCAATTACAATATATACACCAACAAACAATGTTCAACCAGTAACTACTACTAAT
14221 CAACGCCCATAATCATACAAAGCCCCCGCACCAATAGGATCCTCCCGAATCAACCCTGAC
14281 CCCTCTCCTTCATAAATTATTCAGCTTCCTACACTATTAAAGTTTACCACAACCACCACC

14341 CCATCATACTCTTTCACCCACAGCACCAATCCTACCTCCAT**CG**CTAACCCCACTAAAACA
14401 CTCACCAAGACCTCAACCCCTGACCCCCATGCCTCAGGATACTCCTCAATAGCCAT**CG**CT
14461 GTAGTATATCCAAAGACAACCATCATTCCCCCTAAATAAATTAAAAAAACTATTAAACCC
14521 ATATAACCTCCCCCAAAATTCAGAATAATAACACACC**CG**ACCACAC**CG**CTAACAATCAAT
14581 ACTAAACCCCCATAAATAGGAGAAGGCTTAGAAGAAAACCCCACAAACCCCATTACTAAA
14641 CCCACACTCAACAGAAACAAAGCATACATCATTATTCT**CG**CA**CG**GACTACAACCA**CG**ACC
14701 AATGATATGAAAAACCAT**CG**TTGTATTTCAACTACAAGAACACCAATGACCCCAATA**CG**C
14761 AAAACTAACCCCCTAATAAAATTAATTAACCACTCATTCAT**CG**ACCTCCCCACCCCATCC
14821 AACATCTC**CG**CATGATGAAACTT**CG**GCTCACTCCTTGG**CG**CCTGCCTGATCCTCCAAATC
14881 ACCACAGGACTATTCCTAGCCATGCACTACTCACCAGA**CG**CCTCAAC**CG**CCTTTTCATCA
14941 AT**CG**CCCACATCACT**CG**AGA**CG**TAAATTATGGCTGAATCATC**CG**CTACCTTCA**CG**CCAAT
15001 GG**CG**CCTCAATATTCTTTATCTGCCTCTTCCTACACAT**CG**GG**CG**AGGCCTATATTA**CG**GA
15061 TCATTTCTCTACTCAGAAACCTGAAACAT**CG**GCATTATCCTCCTGCTTGCAACTATAGCA
15121 ACAGCCTTCATAGGCTATGTCCTCC**CG**TGAGGCCAAATATCATTCTGAGGGGCCACAGTA
15181 ATTACAAACTTACTATC**CG**CCATCCCATACATTGGGACAGACCTAGTTCAATGAATCTGA
15241 GGAGGCTACTCAGTAGACAGTCCCACCCTCACA**CG**ATTCTTTACCTTTCACTTCATCTTG
15301 CCCTTCATTATTGCAGCCCTAGCAACACTCCACCTCCTATTCTTGCA**CG**AAA**CG**GGATCA
15361 AACAACCCCCTAGGAATCACCTCCCATTC**CG**ATAAAATCACCTTCCACCCTTACTACACA
15421 ATCAAAGA**CG**CCCT**CG**GCTTACTTCTCTTCCTTCTCTCCTTAATGACATTAACACTATTC
15481 TCACCAGACCTCCTAGG**CG**ACCCAGACAATTATACCCTAGCCAACCCCTTAAACACCCCT
15541 CCCCACATCAAGCC**CG**AATGATATTTCCTATT**CG**CCTACACAATTCTC**CG**ATC**CG**TCCCT
15601 AACAAACTAGGAGG**CG**TCCTTGCCCTATTACTATCCATCCTCATCCTAGCAATAATCCCC
15661 ATCCTCCATATATCCAAACAACAAAGCATAATATTT**CG**CCCACTAAGCCAATCACTTTAT
15721 TGACTCCTAGC**CG**CAGACCTCCTCATTCTAACCTGAAT**CG**GAGGACAACCAGTAAGCTAC
15781 CCTTTTACCATCATTGGACAAGTAGCATC**CG**TACTATACTTCACAACAATCCTAATCCTA
15841 ATACCAACTATCTCCCTAATTGAAAACAAAATACTCAAATGGGCCTGTCCTTGTAGTATA
15901 AACTAATACACCAGTCTTGTAAAC**CG**GAGATGAAAACCTTTTTCCAAGGACAAATCAGAG
15961 AAAAAGTCTTTAACTCCACCATTAGCACCCAAAGCTAAGATTCTAATTTAAACTATTCTC
16021 TGTTCTTTCATGGGGAAGCAGATTTGGGTACCACCCAAGTATTGACTCACCCATCAACAA
16081 C**CG**CTATGTATTT**CG**TACATTACTGCCAGCCACCATGAATATTGTA**CG**GTACCATAAATA
16141 CTTGACCACCTGTAGTACATAAAAACCCAATCCACATCAAAACCCCCTCCCCATGCTTAC
16201 AAGCAAGTACAGCAATCAACCCTCAACTATCACACATCAACTGCAACTCCAAAGCCACCC
16261 CTCACCCACTAGGATACCAACAAACCTACCCACCCTTAACAGTACATAGTACATAAAGCC
16321 ATTTAC**CG**TACATAGCACATTACAGTCAAATCCCTTCT**CG**TCCCCATGGATGACCCCCCT
16381 CAGATAGGGGTCCCTTGACCACCATCCTC**CG**TGAAATCAATATCC**CG**CACAAGAGTGCTA
16441 CTCTCCT**CG**CTC**CG**GGCCCATAACACTTGGGGGTAGCTAAAGTGAACTGTATC**CG**ACATC
16501 TGGTTCCTACTTCAGGGTCATAAAGCCTAAATAGCCCACA**CG**TTCCCCTTAAATAAGACA
16561 TCA**CG**
